# Independent Cis-Regulatory Modules within the Herpes Simplex Virus 1 Infected Cell Protein 0 (ICP0) Promoter Are Transactivated by Krüppel-like Factor 15 and Glucocorticoid Receptor

**DOI:** 10.3390/v14061284

**Published:** 2022-06-13

**Authors:** Nishani Wijesekera, Nicholas Hazell, Clinton Jones

**Affiliations:** 1 Department of Veterinary Pathobiology, College of Veterinary Medicine, Oklahoma State University, 208 N McFarland Street, RM 250 McElroy Hall, Stillwater, OK 74078, USA; nwijese@okstate.edu; 2 Experimental Pathology Program, University of Texas Medical Branch, 301 University Blvd, Galveston, TX 77555, USA; nchazell@utmb.edu

**Keywords:** herpes simplex virus type 1 (HSV-1), infected cell protein 0 (ICP0) expression, stress, Krüppel-like factor 15, glucocorticoid receptor (GR)

## Abstract

A corticosteroid antagonist impairs Herpes Simplex Virus 1 (HSV-1) productive infection and explant-induced reactivation from latency, suggesting corticosteroids and the glucocorticoid receptor (GR) mediate certain aspects of these complex virus–host interactions. GR-hormone complexes regulate transcription positively and negatively, in part, by binding GR response elements (GREs). Recent studies revealed infected cell protein 0 (ICP0), ICP4, and ICP27 promoter/cis-regulatory modules (CRMs) are cooperatively transactivated by GR and Krüppel-like factor 15 (KLF15), which forms a feed-forward transcription loop. We hypothesized the ICP0 promoter contains independent CRMs that are transactivated by GR, KLF15, and the synthetic corticosteroid dexamethasone (DEX). This hypothesis is based on the finding that the ICP0 promoter contains multiple transcription factor binding sites, and GR and KLF15 cooperatively transactivate the full-length ICP0 promoter. ICP0 promoter sequences spanning −800 to −635 (fragment A) were efficiently transactivated by GR, KLF15, and DEX in monkey kidney cells (Vero), whereas GR and DEX significantly enhanced promoter activity in mouse neuroblastoma cells (Neuro-2A). Furthermore, ICP0 fragment B (−458 to −635) was efficiently transactivated by GR, KLF15, and DEX in Vero cells, but not Neuro-2A cells. Finally, fragment D (−232 to −24) was transactivated significantly in Vero cells by GR, KLF15, and DEX, whereas KLF15 and DEX were sufficient for transactivation in Neuro-2A cells. Collectively, these studies revealed efficient transactivation of three independent CRMs within the ICP0 promoter by GR, KLF15, and/or DEX. Finally, GC-rich sequences containing specificity protein 1 (Sp1) binding sites were essential for transactivation.

## 1. Introduction

Sensory neurons in trigeminal ganglia (TG) are important sites for herpes simplex virus-1 (HSV-1) latency following acute infection of the oral, ocular, or nasal cavity [1,2]. Primary ocular infections can lead to follicular conjunctivitis, superficial punctate keratitis, and dendritic ulcers. In fact, HSV-1 is the leading cause of infectious blindness worldwide, and there are approximately 50,000 new cases each year. Approximately 20% of patients develop recurrent stromal keratitis, which is the result of reactivation from latency. Furthermore, HSV-1-induced recurrent encephalitis is generally due to reactivation from latency [3]. Hence, the ability of HSV-1 to reactivate from latency is crucial for virus transmission and recurrent eye disease. Identifying cellular factors that trigger productive infection and/or reactivation from latency may yield novel strategies to impair productive infection and reduce the incidence of reactivation from latency.

HSV-1 encodes five immediate early (IE) viral mRNAs that are expressed in the absence of de novo protein synthesis during productive infection: ICP0, ICP4, ICP22, ICP27, and ICP47 [4]. A viral tegument protein (VP16) interacts with two host transcription factors, Oct1 and host cellular factor 1 (HCF1), to transactivate IE gene expression, reviewed in [5,6]. ICP0, a product of the RL2 gene, is a promiscuous activator of promoters, has E3 ubiquitin ligase activity (reviewed in [7,8]), and enhances histone removal and acetylation on viral DNA [9]. ICP0 expression promotes reactivation from latency [10,11], in part, because it impairs innate immune responses during early stages of reactivation [12]. Collectively, ICP0 functions stimulate productive infection.

The incidence of HSV-1 reactivation from latency in humans correlates with increased stress: for example, exposure to UV light, heat stress (fever), and trauma [1,13,14,15,16,17,18]. Stressful stimuli generally increase corticosteroid levels, which enter a cell, and bind to the glucocorticoid receptor (GR) or mineralocorticoid receptor (MR), reviewed in [19]. The MR or GR corticosteroid complex enters the nucleus, specifically binds a glucocorticoid response element (GRE), alters chromatin confirmation, and activates transcription. In response to stress, GR and Krüppel-like transcription factor 15 (KLF15) regulate gene expression by a positive feed-forward loop [20,21,22]. For example, GR activates KLF15 expression, and then, GR directly binds KLF15; consequently, a novel set of genes are synergistically activated by GR and KLF15 when compared to GR or KLF15.

Growing evidence has demonstrated that corticosteroids and KLF15 mediate HSV-1 replication and reactivation from latency, as summarized below. For example, primary human gingival fibroblasts treated with the synthetic corticosteroid dexamethasone (DEX) prior to HSV infection yielded significantly higher levels of the virus relative to controls only infected with HSV-1 [23]. This study further revealed that infection increased nuclear GR levels after infection when compared to controls. Our recent studies indicated HSV-1 infection of a mouse neuroblastoma cell line (Neuro-2A) that was treated with DEX also yield higher levels of the infectious virus [24]. Furthermore, the corticosteroid-specific antagonist, CORT-108297, significantly reduces HSV-1 replication in Neuro-2A cells, but has no effect on cell viability [24]. We and others demonstrated that DEX accelerates explant-induced reactivation from latency, as judged by increased virus production [25,26,27]. In accordance with the findings that DEX promotes explant-induced reactivation from latency, CORT-108297 significantly reduces virus shedding during explant-induced reactivation [27]. Finally, HSV-1 induces (KLF15) steady state protein levels during productive infection, and silencing KLF15 significantly reduces viral replication [28]. Collectively, these compelling studies indicate GR and DEX promote viral replication and reactivation from latency.

To probe the mechanism by which GR and stress stimulate viral replication and reactivation from latency, we tested whether GR and stress-induced cellular transcription factors transactivate key viral promoters. These studies demonstrated that HSV-1 IE promoter/cis-regulatory modules (CRMs) that drive expression of key viral transcriptional regulators (ICP0, ICP4, and ICP27) are cooperatively transactivated by GR and KLF15 [24,29,30]. Surprisingly, the ICP0, ICP4, and ICP27 promoters do not contain consensus GREs. KLF family members and specificity protein 1 (Sp1) belong to the same super family of transcription factors, and these family members bind GC-rich sequences [31,32]. Mutagenesis studies suggest Sp1 and/or GC rich motifs are important for cooperative transactivation of HSV-1 IE promoters [24,29,30]. These studies further imply interactions between GR and KLF15 overcome the requirement that GR must bind a consensus GRE. Understanding how stress-induced transcription factors, including GR and KLF15, trigger HSV-1 gene expression is important because the viral genome exists as silent chromatin during latency [33,34]. Since viral transcriptional regulatory proteins are not abundantly expressed in latently infected cells, it is reasonable to suggest that cellular transcription factors trigger viral gene expression following stressful stimuli.

These studies were focused on identifying CRMs upstream of the ICP0 TATA box that increase transcription of a heterologous minimal promoter; test whether CRMs are cooperatively transactivated by GR, KLF15, and/or DEX; and identify CRM sequences important for transactivation. These studies provide new insight into how GR and KLF15 increase ICP0 expression and productive infection.

## 2. Materials and Methods

### 2.1. Cells

Mouse neuroblastoma cells (Neuro-2A, ATCC CCL-131) and monkey kidney epithelial cells (Vero, ATCC, CCL-81) were grown in MEM with 2 mM L-glutamine, 100 mg/mL Streptomycin, 10 U/mL penicillin, and 10% fetal bovine serum (FBS) (Atlas Biologicals; Fort Collins, CO, USA, EF-0500-A), as previously described [24,25]. Activated charcoal selectively removes lipophilic molecules, including corticosteroids that drive cellular stress responses, without affecting salts, glucose, or amino acids. This allows for improved control over glucocorticoid receptor activation with specific concentrations of DEX.

### 2.2. Dual Luciferase Assay

Neuro-2A and Vero cells were cultured in 60 mm dishes until 80% confluency. Two hours before transfection, cells were washed with PBS and antibiotic free media with 2% charcoal stripped FBS (Sigma-Aldrich; St Louis, MO, USA, 12676029) added. Cells were transfected with pGL4.24[luc2/minP] luciferase reporter plasmid (Promega; Madison, WI, USA; E842A) containing one of the ICP0 CRMs inserted upstream of the minimal promoter (0.5 ug DNA), and a plasmid expressing Renilla luciferase (0.05 ug DNA), from a minimal TK promoter as a transfection control. Where indicated, GR-α (1 ug DNA) and KLF15 (0.5 ug DNA) expression plasmids were co-transfected with the reporter plasmid. Empty vector plasmid was added as needed to maintain equal quantities of DNA in all transfection reactions. Transfection was carried out using Lipofectamine 3000 (Invitrogen, catalog no. L3000075, Waltham, MA, USA) following manufacturer’s instructions. At 24 h following transfection, water soluble DEX (Sigma; St Louis, MO, USA; D2915; 10 uM final concentration) was added to designated samples. At 48 h post-transcription, cells were washed, harvested using passive lysis buffer, and stored at −80 °C. Lysate was subjected to a dual luciferase assay, as previously described [24,25]. Firefly and Renilla luciferase activity in each sample were measured using a commercially available kit (Promega; Madison, WI, USA; E1910) and a Glomax 20/20 luminometer (Promega; Madison, WI, USA; E5331). Promoter activity in the pGL4.24[luc2/minP] luciferase reporter plasmid was calculated as a ratio of the reporter firefly luciferase to Renilla luciferase activity, the transfection control. Activity of the empty pGL4.24[luc2/minP] plasmid transfected alone was set as the promoter baseline. Increased promoter activity over this baseline was calculated as fold activation induced by an ICP0 CRM construct and transactivation by GR, KLF15, and DEX treatment. 

#### 2.2.1. Plasmids

The full-length ICP0 promoter (−800 to +150) was previously described, and provided by the late Dr. Priscilla Schafer [35]. The respective CRM constructs were synthesized by Genscript and inserted into pGL4.23[luc2/minP] (Promega; Madison, WI, USA) at SacI and XhoI unique restriction enzyme sites. The GR-α expression construct was obtained from Dr. John Cidlowski (NIEHS, Research Triangle Park, NC, USA). The KLF15 expression plasmid was obtained from Deborah Otteson (University of Houston; Houston, TX, USA).

#### 2.2.2. Statistical Analysis

All graphs and analyses were made using Microsoft Excel. Student’s *t*-test with two-tailed and two-sample unequal variance was performed on the data sets. An * denotes a significant difference in construct alone compared to its own co-transfected samples. A # denotes a significant difference in mutant samples compared to the relevant wt CRM construct. The */# denotes a significant difference of *p* < 0.05, and **/## denotes a significant difference of *p* < 0.005, as determined by Student’s *t*-test. NS denotes a non-significant difference.

## 3. Results

### 3.1. GR and KLF15 Transactivate Specific ICP0 Promoter CRM Fragments

**Figure 1:**
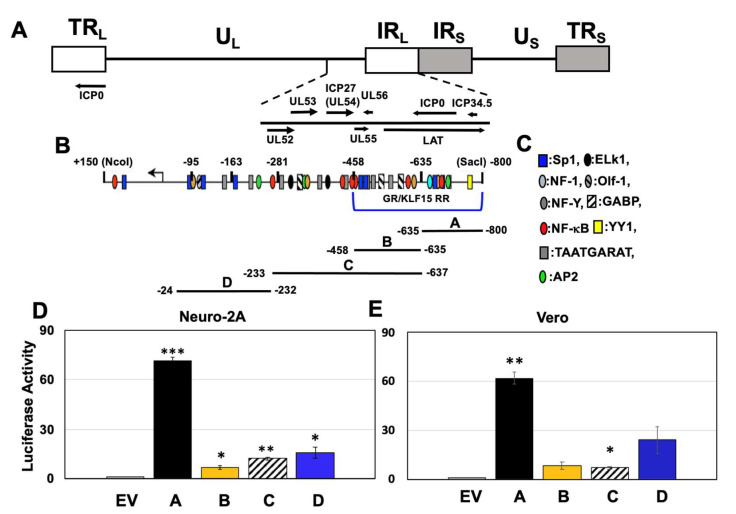
**Location of ICP0 gene and promoter/enhancer sequences within the IR_L_ and TR_L_ repeats of the HSV**−**1 genome**. (**Panel A**): Schematic of HSV-1 genome. Unique long (U_L_) and unique short (U_S_) segments are flanked by the long internal or terminal repeats (IR_L_ and TR_L_: white rectangles) and short internal or terminal (IR_S_ and TR_S_: gray rectangles). Location of known genes in IR_L_, including ICP0, are shown. A copy of ICP0 is also present in the TR_L_. (**Panel B**): Schematic of ICP0 promoter, and location of potential transcription factor binding sites (**Panel C**) relative to the start site of ICP0 mRNA (arrow). Four fragments (**A**–**D**) were used in this study and were cloned upstream of the pGL4.24[luc2/minP] firefly luciferase reporter plasmid. Neuro-2A (**Panel D**) or Vero (**Panel E**) cells were transfected with 0.5 ug of the denoted CRM constructs and a Renilla luciferase plasmid (0.05 ug). At 48 h after transfection, dual luciferase activity was performed. Fold activation was calculated relative to pGL4.24[luc2P/minP] or empty vector (EV) for each construct. Results are the mean of three experiments, and error bars indicate standard error. The *, ** and *** denote a significant difference of *p* < 0.05, *p* < 0.005 and *p* < 0.0005 respectively compared to the EV luciferase activity as determined by Student’s *t*-test. Statistical analysis was performed as described in Materials and Methods.

The ICP0 gene is located within the long repeats (TR_L_ and IR_L_) of the HSV-1 genome (Figure 1A), and the ICP0 promoter contains numerous putative transcription factor binding sites [35] (Figure 1B,C). Previous studies identified a GR/KLF15 responsive region (RR) at the 5′-terminus of the ICP0 promoter (−800 to −458; Figure 1B) [24]. To identify ICP0 non-coding sequences that can stimulate transcription, four ICP0 fragments upstream of the TATA box were synthesized (Figure 1B) and cloned at the 5′-terminus of a minimal promoter in the luciferase reporter construct (pGL4.24[luc2P/minP]).

Transcriptional activity was measured in a mouse neuroblastoma (Neuro-2A) and monkey kidney cell line (Vero). The rationale for using these two cell lines is that they can be readily transfected, and Vero cells are highly permissive for HSV-1 infection. Furthermore, Neuro-2A cells are neuronal-like cells that can be differentiated into dopaminergic-like neurons [36]. Neuro-2A cells express low levels of GR and KLF15 [37]. Silencing KLF15 significantly impairs HSV-1 replication [28], and the corticosteroid-specific antagonist (CORT-108297) significantly reduces HSV-1 replication [24] in Neuro-2A cells. The endogenous GR in Neuro-2A cells is smaller than the protein produced by the GR-α expression plasmid used for transient transfection studies, and the endogenous GR does not transactivate HSV-1 as efficiently as when GR-α is expressed [24,29,30] (data not shown). GR receptor isoforms, generated by alternative splicing, do not have a transcriptional activation potential as high as GR-α [38], suggesting the GR expressed in Neuro-2A cells is an isoform that does not activate gene expression as efficiently as GR-α, or that the GR gene has a deletion. A GR-specific band migrating with similar mobility as GR-α and a smaller band are expressed at low levels in Vero cells (data not shown), and GR-α transactivates HSV-1 promoters more efficiently than the endogenous GR protein [24,29,30]. In general, there is no selective pressure to express GR at high levels in established cell lines because GR expression can induce apoptosis and cell cycle arrest in certain cells, reviewed in [19,39]. The A fragment consistently activated the minimal promoter approximately 60-fold in Neuro-2A (Figure 1D) and Vero cells (Figure 1E). The B fragment luciferase construct exhibited significantly higher than pGL4.24[luc2P/minP] in Neuro-2A, but not in Vero cells. Though the CRM activity of fragment C was less than 10-fold higher in Vero cells compared to the empty vector, it was significantly higher than the empty vector. Since fragment C exhibited similar activity as fragment B, we did not pursue additional studies with C because sequences in fragment B were primarily responsible for CRM activity. The fragment D construct was not significantly different than the empty vector in Vero cells because it yielded variable results. In summary, these studies revealed the ICP0 promoter contained independent CRMs, and the GR/KLF15 RR contained two fragments with CRM activity.

### 3.2. Localization of Fragment A CRM Activity

**Figure 2:**
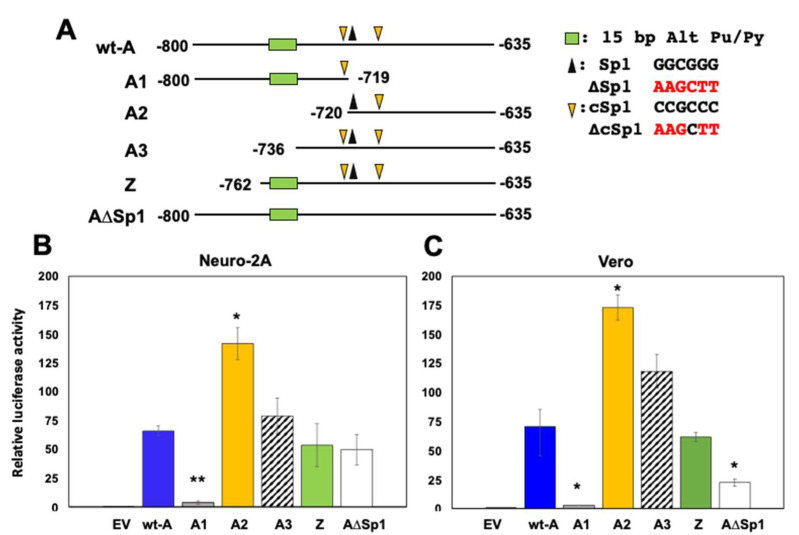
**Localization of ICP0 fragment Athat exhibits CRM activity.** (**Panel A**): Schematic of ICP0 wt−A fragment with transcription factor binding sites and mutants used in the study. A1 and A2 contain the 5′ half and 3′ half of the wt-A fragment. Fragment A3 contains all Sp1 and cSp1 binding sites but lacks the 15−base alternating purine/pyrimidine motif. Fragment Z contains the alternating purine/pyrimidine motif (CGCGCATATATACGCTTG) and all Sp1/cSp1 binding sites. The A∆Sp1 construct has mutations in all Sp1/cSp1 binding sites. All constructs were cloned into the pGL4.24[luc2P/minP] vector such that the fragment is upstream of the minimal promoter. Nucleotide position numbers shown are relative to the ICP0 mRNA initiation site. Nucleotide sequence of Sp1 and cSp1 sites (black) and mutants (red) are shown to the right of panel A. Neuro−2A (**Panel B**) or Vero (**Panel C**) cells were transfected with 0.5 µg with the wt A construct or designated A mutant and Renilla luciferase plasmid (0.05 µg). 48 h after transfection, cells were harvested, and dual luciferase assay was performed. Luciferase activity was calculated relative to pGL4.24[luc2P/minP] for each construct. Results are the mean of three experiments, and the error bars indicate standard error. The * and ** denote a significant difference of *p* < 0.05 and *p* < 0.005 respectively compared to the wt-A CRM luciferase activity as determined by Student’s *t*-test. Statistical analysis was performed as described in Materials and Methods.

Since fragment A exhibited the highest cis-activation of the luciferase construct, additional sub-fragments were synthesized, and transcriptional activity was measured. Fragment A contains a Sp1 binding site (GGCGGG), two complements of a consensus Sp1 binding site (denoted cSp1: CCGCCC), and a 15-base alternating purine/pyrimidine motif, which has the potential to form Z-DNA [40] (Figure 2A). The Sp1 and cSp1 binding sites have the potential to form a stem-loop structure, suggesting secondary structures exist in fragment A. Previous studies demonstrated that mutating certain Sp1 sites within the ICP4 and ICP27 enhancers reduced the ability of GR and KLF15 to transactivate these promoters [29,30], suggesting Sp1 or cSp1 sites were important for the CRM activity of fragment A. Surprisingly, fragment A2 exhibited significantly higher levels of CRM activity relative to intact fragment A in Neuro-2A and Vero cells (Figure 2B,C). However, fragment A1 had significantly lower CRM activity relative to wt-A fragment. Fragments A3 and Z were not significantly different than wt-A fragment in Neuro-2A or Vero cells, suggesting the Z DNA motif had little effect on transcriptional activity. Interestingly, transcriptional activity of fragment A∆Sp1 was significantly reduced in Vero, but not in Neuro-2A cells. In summary, these studies revealed that sequences encompassing −720 to −635 (fragment A2) exhibited stronger transcriptional activity than wt-A and Sp1, plus cSp1 binding sites were crucial for stimulating transcriptional activity in Vero cells.

### 3.3. Localization of Fragment A Sequences That Mediate Transactivation by GR, KLF15, and/or DEX

**Figure 3:**
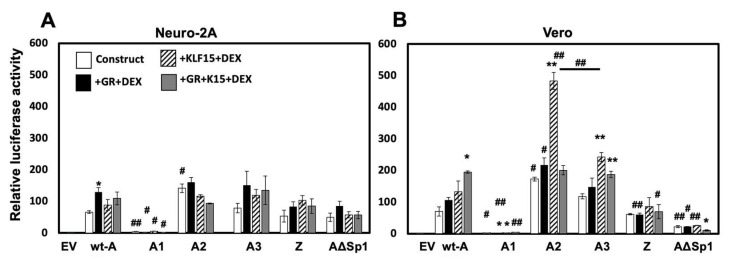
**Identification of sequences in ICP0 fragment A that mediate transactivation by GR, KLF15, and/or DEX.** Neuro-2A (**Panel A**) or Vero (**Panel B**) cells were transfected with 0.5 µg of the wt A luciferase construct, or the designated A mutant fragments and Renilla luciferase expression plasmid (0.05 µg), as described in Materials and Methods. Where indicated, the denoted cultures were co-transfected with GR (1 ug) and/or KLF15 (0.5 ug) expression plasmids. Empty vector (pGL4.24[luc2P/minP]) was added to cultures to maintain the same DNA amount. 24 h after transfection, certain cultures were treated with DEX (10 uM). Cells were harvested at 48 h, and dual luciferase assay was performed. Luciferase activity was calculated relative to empty vector (EV) luciferase value. Results are the mean of three experiments, and error bars denote the standard error. An * denotes a significant difference compared to the luciferase construct alone. A # denotes a significant difference in the denoted mutant construct relative to the respective wt A transfected samples. A */# denotes a significant difference of *p* < 0.05, whereas a **/## denotes a significant difference of *p* < 0.005, as determined by Student’s *t*-test.

The ability of GR, KLF15, and/or DEX to transactivate the wt-A fragment and the respective mutants (Figure 2A) was examined in Neuro 2A and Vero cell lines. The wt-A fragment was significantly transactivated by GR and DEX in Neuro-2A cells; however, DEX and KLF15 addition did not further increase promoter activity (Figure 3A). Conversely, the wt-A fragment was significantly transactivated by GR, KLF15, and DEX treatment in Vero cells (Figure 3B). Fragment A1 was not transactivated by GR and/or KLF15 in both cell lines regardless of DEX treatment, confirming this fragment inhibits promoter activity. In Neuro-2A cells, fragments A2 and A3 were not significantly transactivated by GR, KLF15, and/or DEX treatment when compared to the wt-A fragment. Conversely, fragment A2 was significantly transactivated by GR or KLF15 in Vero cells when compared to the wt-A fragment but adding GR together with KLF15 and DEX treatment reduced KLF15- and DEX-mediated transactivation. Fragment A3 exhibited reduced transactivation by KLF15 and DEX when compared to the A2 fragment in Vero cells. In both cell lines, the ability of GR and KLF15 to transactivate fragment Z was not higher than the wt-A fragment.

The A∆Sp1 fragment, which contains mutations in the Sp1 and cSp1 binding sites of fragment A, was compared to the wt-A fragment for transactivation by GR, KLF15, and/or DEX in Vero and Neuro-2A cells. The A-∆Sp1 construct was not significantly transactivated by GR and KLF15 regardless of DEX treatment in Neuro-2A cells. Transactivation of the A-ΔSp1 mutant by GR, KLF15, and DEX treatment in Vero cells was significantly reduced when compared to wt fragment A. In contrast to the results in Vero cells, the A-∆Sp1 construct did not dramatically reduce luciferase activity relative to the wt-A or A2 fragment in Neuro-2A cells. Collectively, these studies revealed cell-type-dependent effects were observed with respect to transactivation of the wt-A, A2, and A∆Sp1 fragments in Vero versus Neuro-2A cells.

### 3.4. Sp1 and cSp1 Binding Sites in the B Fragment Mediate CRM Activity

**Figure 4:**
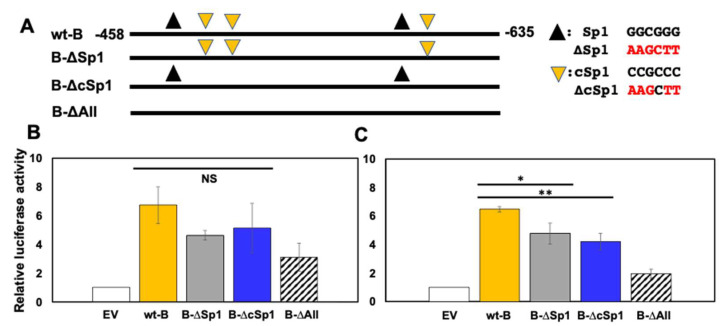
**Localization of ICP0 B fragmentCRM activity.** (**Panel A**): ICP0 wt-B fragment, location of Sp1 and cSp1 binding sites, and fragment B mutants are shown. Nucleotide position numbers are relative to the transcription initiation site of ICP0 mRNA. All constructs were cloned into pGL4.24[luc2P/minP]. The nucleotide sequence of Sp1, cSp1 sites, and their mutated form (red) is shown to the right of the figure. Neuro-2A (**Panel B**) or Vero (**Panel C**) cells were incubated in 2% stripped FBS and transfected with 0.5 ug of wt-B construct or denoted B mutant construct and a Renilla luciferase plasmid (0.05 ug). 48 hrs after transfection, cells were harvested, and dual luciferase assay was performed. Fold activation was calculated relative to pGL4.24[luc2P/minP] or empty vector (EV) for each construct. Results are the mean of three experiments, and error bars denote standard error. The * and ** denote a significant difference of *p* < 0.05 and *p* < 0.005 respectively compared to the wt-B CRM luciferase activity as determined by Student’s *t*-test. Statistical analysis was performed as described in Materials and Methods.

Although CRM activity of the wt-B fragment was modest, it was examined further because it contains two consensus Sp1 binding sites and two cSp1 binding sites (Figure 4A), and is contained within the GR/KLF15 RR of the ICP0 promoter (Figure 1B). Since Sp1 and certain GC rich motifs that resemble Sp1 binding sites are crucial for GR-, KLF15-, and/or DEX-mediated transactivation [29,30], mutants of both Sp1 (B-∆Sp1) and cSp1 (B-∆cSp1), or Sp1 and cSp1 binding sites (B∆All) were prepared. In Vero (Figure 4C), but not Neuro-2A, cells (Figure 4B), all three fragment B mutants exhibited significantly reduced transcriptional activity when compared to the-wt B fragment. Notably, the B∆All construct exhibited the least CRM activity, suggesting Sp1 and cSp1 binding sites were important for stimulating transcription in Vero cells. Although wt-B and the three mutants did not have significantly different CRM activity in Neuro-2A cells due to variability in the individual results, the three mutant constructs exhibited reduced transcriptional activity, with B∆All having the lowest activity.

### 3.5. Sp1 Binding Sites Mediate Transactivation of B Fragment by GR, KLF15, and DEX In Vero Cells

**Figure 5:**
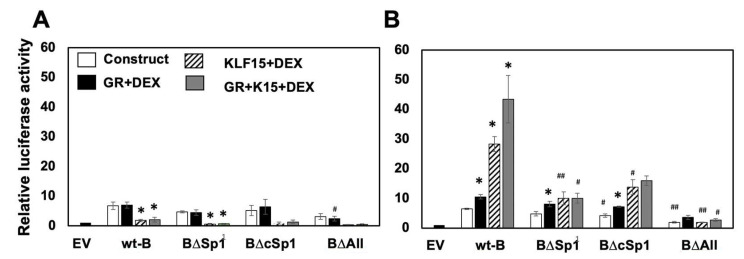
**Transactivation of fragment B and mutants by GR, KLF15, and/or DEX.** Neuro-2A (**Panel A**) or Vero (**Panel B**) cells were incubated in 2% stripped FBS and transfected with 0.5 ug of the denoted B constructs and a Renilla luciferase plasmid (0.05 ug). Denoted samples were co-transfected with GR (1 ug) and/or KLF15 (0.5 ug) expression plasmids. Empty vector (pGL4.24[luc2P/minP]) was added to cultures to maintain the same DNA concentration. 24 h after transfection, certain cultures were incubated with water-soluble DEX (10 uM). Cells were harvested at 48 h, and dual luciferase assay was performed. Luciferase activity was calculated relative to empty vector luciferase activity. Results are the mean of three experiments, and error bars indicate standard error. An * denotes a significant difference compared to the luciferase construct alone. A # denotes a significant difference in the denoted mutant construct relative to the respective wt B transfected samples. A */# denotes a significant difference of *p* < 0.05, whereas a ## denotes a significant difference of *p* < 0.005, as determined by Student’s *t*-test. Statistical analysis was performed as described in Materials and Methods.

In Neuro-2A cells, the wt-B fragment was not readily transactivated by GR, KLF15, and/or DEX (Figure 5A). Surprisingly, there was a significant reduction of promoter activity when transfected with KLF15 + DEX or GR + KLF15 + DEX. These results were consistent with the finding that Sp1 and cSp1 binding sites in the wt-B fragment did not significantly reduce transcriptional activity in Neuro-2A cells.

In contrast to Neuro-2A cells, the wt-B fragment was significantly transactivated by GR and DEX or KLF15 and DEX or GR, KLF15 and DEX in Vero cells (Figure 5B). These studies also revealed that the transactivation of fragment B was significantly reduced when Sp1 binding sites (B∆Sp1) or cSp1 binding sites (B∆cSp1) were mutated and then transfected with GR, KLF15, and/or DEX. When all Sp1 and cSp1 binding sites were mutated, promoter activity was at basal levels following transfection with GR, KLF15, and/or DEX. Collectively, these studies indicated that Sp1 and cSp1 binding sites were crucial for transactivation of the fragment B by GR, KLF15, and DEX in Vero cells.

### 3.6. Sp1 Binding Sites in Fragment D are Necessary for Stimulating Transcription In Vero Cells

**Figure 6:**
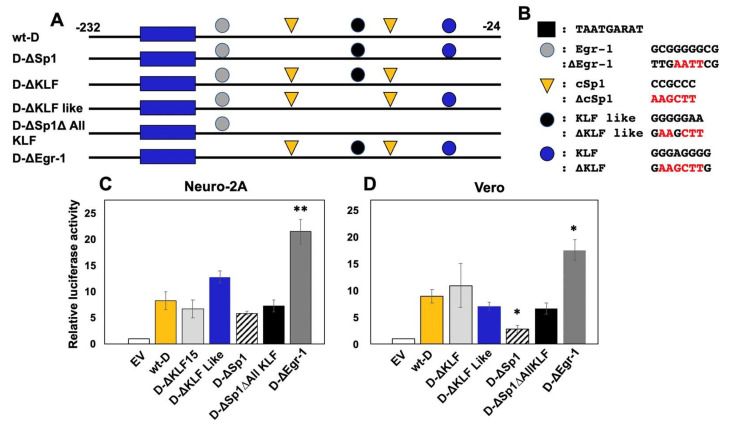
**Examination of transcriptional activation of a minimal promoter by fragment D.** (**Panel A**): ICP0 wt−D fragment, location of transcription factor binding sites, and schematic of the respective mutants are shown. All constructs were cloned upstream of the minimal promoter of pGL4.24[luc2P/minP]. Nucleotide position numbers are shown relative to the transcription initiation site of ICP0 mRNA. (**Panel B**): nucleotide sequences of Sp1, KLF, KLF-like, and Egr−1 sites, and the respective mutants (red) are shown. Neuro−2A (**Panel C**) or Vero (**Panel D**) cells were incubated in 2% stripped FBS and transfected with 0.5 ug of the wt−D construct or denoted mutants and Renilla luciferase expression plasmid (0.05 ug). 48 hrs after transfection, cells were harvested, and dual luciferase assays were performed. Luciferase activity was calculated relative to pGL4.24[luc2P/minP] for each construct. Results are the mean of three experiments, and error bars indicate standard error. The * and ** denote a significant difference of *p* < 0.05 and *p* < 0.005 respectively compared to the wt−D CRM luciferase activity as determined by Student’s *t*−test. Statistical analysis was performed as described in Materials and Methods.

To identify sequences within fragment D that possess CRM activity, wt-D, and the respective D mutants (Figure 6A,B) were transfected into Neuro-2A or Vero cells, and transcriptional activity was measured. The wt-D fragment consistently stimulated promoter activity 5–10-fold higher than the empty luciferase reporter construct in Neuro-2A (Figure 6C) and Vero cells (Figure 6D). Within fragment D, we identified a motif similar to a consensus Egr-1 binding site (GCGGGGGCG) [41]. Egr-1 binding sites downstream of the HSV-1 and HSV-2 start sites of VP16 transcription were reported to enhance virulence and reactivation from latency [42]. However, binding of Egr-1 to a consensus binding site in its own promoter or the type II collagen promoter reduces gene expression [41,43]. Mutating the Egr-1-like site in fragment D (D-∆Egr-1) significantly increased CRM activity in Neuro-2A and Vero cells. Mutating the two Sp1 binding sites also significantly reduced transcriptional activity in Vero, but not Neuro-2A, cells. The KLF or KLF-like sites in fragment D did not significantly reduce transcriptional activity in Neuro-2 or Vero cells.

### 3.7. Cell-Type Dependent Transactivation of Fragment D by GR, KLF15, and DEX

**Figure 7:**
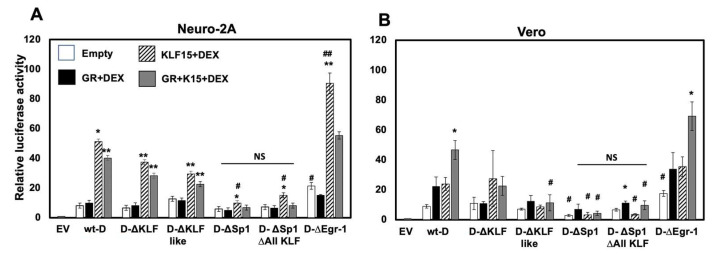
**Transactivation of fragment D constructs and mutants by GR, KLF15, and/or DEX.** Neuro-2A (**Panel A**) or Vero (**Panel B**) cells were incubated in 2% stripped FBS, and transfected with 0.5 ug of wt-D construct or denoted mutant constructs and Renilla luciferase plasmid (0.05 ug). Where indicated, samples were co-transfected with GR (1 ug) and/or KLF15 (0.5 ug) expression plasmids. Empty vector (pGL4.24[luc2P/minP]) was added to cultures to maintain the same DNA amount. 24 h after transfection, certain cultures were treated with DEX (10 uM) as denoted. Cells were harvested at 48 h, and dual luciferase assays were performed. Luciferase activity was calculated relative to the empty vector luciferase activity. Results are the mean of three experiments, and error bars denote standard error. An * denotes a significant difference in a luciferase construct when compared to the luciferase construct cotransfected with GR and/or KLF15 and treated with DEX. A # denotes a significant difference in the mutant luciferase construct compared to relevant wt D transfected samples. A */# denotes a significant difference at *p* < 0.05, whereas a **/## denotes a significant difference as *p* < 0.005, as determined by Student’s *t*-test. NS denotes a non-significant difference between the denoted constructs.

KLF15 and DEX significantly transactivated the wt-D fragment in Neuro-2A cells (Figure 7A); however, GR did not significantly increase promoter activity. Conversely, significant transactivation of wt-D fragment only occurred when Vero cells were transfected with GR, KLF15, and cultures treated with DEX (Figure 7B). Mutating Sp1 binding sites significantly reduced KLF15- and DEX-mediated transactivation in Neuro-2A cells. GR-, KLF15-, and DEX-mediated transactivation of fragment D was also significantly reduced in Vero cells when Sp1 binding sites were mutated. Mutating putative KLF or KLF-like binding sites significantly reduced GR-, KLF15-, and DEX-mediated transactivation in Vero, but not Neuro-2A, cells. Mutating the Egr-1-like binding site significantly enhanced the ability of KLF15 and DEX to transactivate the wt-D fragment construct in Neuro-2A cells. Although mutating the Egr-1 binding site significantly increased GR-, KLF15-, and DEX-mediated transactivation in Vero cells, the effects were not significant compared to the wt-D fragment.

## 4. Discussion

In this study, we present evidence that three distinct CRMs in the ICP0 promoter were transactivated by GR, KLF15, and or DEX. Surprisingly, fragment A contains sequences (−800 to −719) that significantly reduced transcriptional activity in Neuro-2A and Vero cells. Furthermore, mutagenesis of an Egr-I-like binding site in fragment D significantly increased CRM activity in Neuro-2A and Vero cells. Transactivation by GR, KLF15, and/or DEX treatment was also increased in these respective ICP0 CRM fragments when negative regulatory sequences were deleted or mutated. Though it was surprising to identify negative regulatory sequences in ICP0 CRM sequences, we suggest they mediate transcriptional activity during productive infection, promote establishment and maintenance of latency, or enhance ICP0 expression in certain cell types.

**Figure 8:**
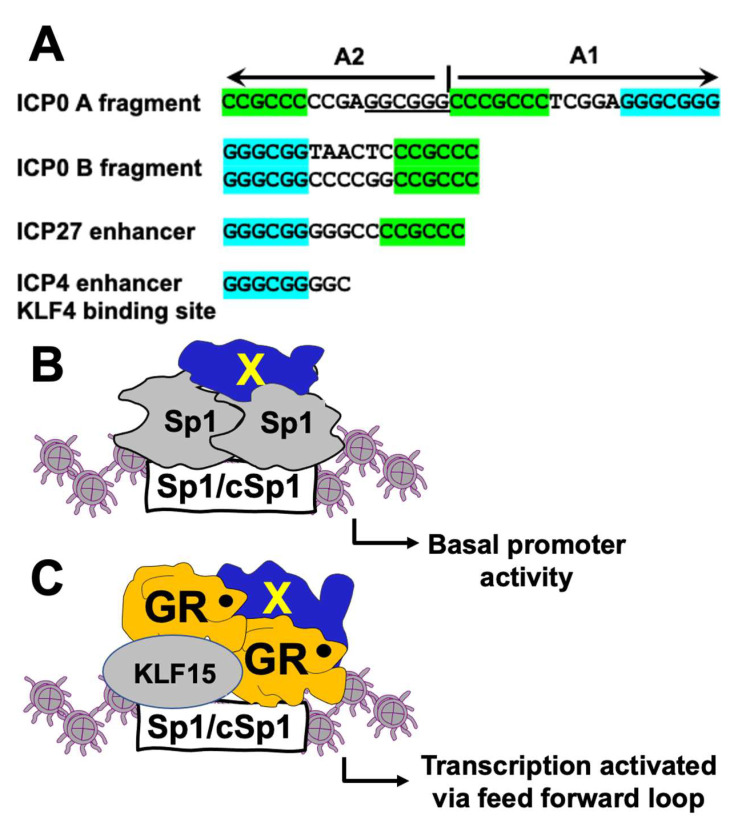
**Summary of GR/KLF15-mediated transactivation of Sp1/cSp1 binding sites in HSV-1 IE promoters and putative transcription factor binding models**. (**Panel A**): Location of Sp1 (GGGCGG), cSp1 sites (CCGCCC), or a Sp1-like motif (GGCGGG) in ICP0 A or B fragments, and the ICP27 enhancer [29]. ICP4 enhancer sequences contain KLF4 consensus binding sites important for KLF4- or KLF15- and GR-mediated transactivation. The KLF4 binding site also contains an Sp1 binding site [30]. (**Panel B**): Model depicting that Sp1 occupies Sp1 and cSp1 binding sites. X are unknown transcriptional coactivators. These interactions lead to basal promoter activity. (**Panel C**): Putative model depicting a complex that contains GR, KLF15, and DEX (black circle associated with GR), and unknown transcriptional coactivators (denoted by X) are bound to Sp1 and/or cSp1 binding site following a stressful stimulus. These interactions culminate in transcriptional activation. Grey circles denote histones.

We [37] and others [22] demonstrated GR and KLF15 stably interact with each other and form a feed-forward transcription loop [20,22]. Consensus GREs are not present in any of the ICP0 fragments that stimulated transcription, suggesting the GR/KLF15 complex interacts with Sp1 or cSp1 binding sites. ICP0 fragments A, B, and D contain Sp1 or cSp1 binding sites crucial for GR-, KLF15-, and/or DEX-mediated transactivation in Vero cells and, to a lesser extent, in Neuro-2A cells. Interestingly, ICP0 fragments A and B contain adjacent cSp1 and Sp1 binding sites separated by 4–6 nucleotides (Figure 8A). Fragment A2, which contains a cSp1 and Sp1-like binding site (GGCGGG), was strongly transactivated by KLF15 and DEX relative to the intact fragment A in Vero cells.

Fragment A1, which contains a distinct Sp1-like and cSp-1-like motif, was not transactivated by GR, KLF15, and/or DEX, suggesting that, under certain circumstances, adjacent sequences can have positive or negative effects on transcription. Recent studies also identified a Sp1 and cSp1 site within HSV-1 ICP27 promoter sequences that is crucial for GR-, KLF15-, and DEX-mediated transactivation [29]. Though the ICP4 enhancer is strongly transactivated by GR, KLF4 or KLF15, and DEX, these sequences do not contain a cSp1 [30]. However, two KLF4 consensus binding sites that encompass a consensus Sp1 site are located in the ICP4 enhancer (Figure 8A). Mutation of the 3′ KLF4 binding site had the same effect as mutating both sites with respect to GR-, KLF4- or KLF15-, and DEX-mediated transactivation in Neuro-2A and Vero cells. ChIP studies generally revealed a correlation between reduced GR occupancy to mutant Sp1 and/or cSp1 sites and transcriptional activation of ICP4 [30] and ICP27 [29] regulatory sequences. In the absence of stressful stimuli, Sp1 and other transcriptional coactivators (denoted by X) are predicted to occupy Sp1 and cSp1 binding sites, which results in basal levels of transcription (Figure 8B). Following a stressful stimulus, GR is activated and KLF15 expression is induced. Consequently, the GR/KLF15 complex is predicted to occupy Sp1 and/or cSp1 binding sites instead of Sp1; thus, transcription is significantly increased (Figure 8C). This model also predicts GR and KLF15 interact with cell-type-specific transcriptional coactivators (denoted as X) that play a role in transcriptional activation. We predict that the levels of GR, KLF15, and other transcriptional regulators in these two cell lines are important with respect to the differences we have observed. Studies designed to test whether the GR/KLF15 complex displaces Sp1 and whether there are other Sp1 family members bound to these sites will provide insight into how GR and KLF15 activate ICP0 promoter activity. It is also of interest to compare whether GR- and KLF15-mediated transactivation of the ICP0, ICP4, and ICP27 CRMs utilize the strategy outlined Figure 8B and C. Finally, not all GC rich motifs are transactivated by GR and KLF15, because mutating the GC-rich Egr-1 like motif significantly increased GR- and KLF15-mediated transactivation of fragment D in Neuro-2A and Vero cells.

Under certain circumstances, DEX does not stimulate GR- and/or KLF15-mediated transactivation of the ICP0 fragments or the ICP4 enhancer [30]. For example, DEX generally enhanced GR- and KLF15-mediated transactivation of the ICP0 fragments in Vero cells, but not Neuro-2A, cells. However, transactivation of fragment A2 by GR and KLF15 was significantly reduced by DEX treatment in Vero cells. Although GR is generally activated by increased hormone levels (ligand-dependent activation), GR can also be activated when corticosteroids are not increased (ligand-independent activation). For example, GR phosphorylation is induced by UV light, which correlates with GR-mediated transcriptional activation and induction of certain enzymes, but corticosteroid levels are not increased [44,45,46]. This observation is intriguing because UV light increases the incidence of HSV-1 and HSV-2 reactivation from latency [16,47,48]. Unliganded GR also increases expression of a tumor suppressor gene, BRCA, via the β-subunit of the ETS transcription factor GA-binding protein [49]. Thirdly, unliganded GR activation increases involucrin expression, but is impaired when the c-Jun N-terminal kinase (JNK) and extracellular signal-regulated kinases (ERK) are inhibited in keratinocytes [50]. Finally, ethanol can increase GR-mediated transcription in the absence of increased corticosteroid levels [51]. Regardless of the ligand-independent stimulus that activates GR, phosphorylation of certain GR serine or threonine residues must occur [44]. Hence, GR-mediated activation of HSV-1 and bovine herpesvirus 1 (BoHV-1) gene expression and productive infection [37,52,53] can be stimulated by ligand-dependent and independent mechanisms.

## 5. Conclusions

These studies provide new insight into the complexity of ICP0 promoter activation, and how a feed-forward transcription loop (GR and KLF15) transactivates the full-length ICP0 promoter. Sp1 and cSp1 sites located in CRMs within ICP0 sequences were crucial for GR-, KLF15-, and/or DEX-mediated transactivation. Since ICP0 encodes multiple functions that support productive infection and reactivation from latency, understanding how stressful stimuli trigger ICP0 promoter activity is important. Notably, these studies revealed that mutating just one important cis-acting motif in the full-length ICP0 promoter may not abolish its ability to be transactivated by stressful stimuli: for example, GR, KLF15, and/or DEX.

## Data Availability

Not applicable.
